# A review of neonatal peripherally inserted central venous catheters in extremely or very low birthweight infants based on a 3-year clinical practice: Complication incidences and risk factors

**DOI:** 10.3389/fped.2022.987512

**Published:** 2022-10-31

**Authors:** Yaohua Wu, Jing Yan, Mengyan Tang, Yanling Hu, Xingli Wan, Xiaowen Li, Qiong Chen, Xia Li

**Affiliations:** ^1^ Department of Neonatology Nursing, West China Second University Hospital, Sichuan University/West China School of Nursing, Sichuan University, Chengdu, China; ^2^ Key Laboratory of Birth Defects and Related Diseases of Women and Children (Sichuan University), Ministry of Education, Chengdu, China; ^3^ Department of Child Healthcare Nursing, West China Second University Hospital, Sichuan University/West China School of Nursing, Sichuan University, Chengdu, China

**Keywords:** catheterization, peripheral, infant, low birthweight, complications, catheterization, peripheral, infant, low birthweight, complications

## Abstract

**Background:**

The application of peripherally inserted central venous catheters (PICCs) in neonates has proven effective in avoiding repetitive insertions and excessive use of transfusion consumables. However, the frequent occurrence of PICC-associated complications deserves special attention, especially in extremely or very low birthweight (E/VLBW) infants, which in turn affects the quality of neonatal PICC practice. Therefore, we conducted a retrospective study of a 3-year clinical practice of neonatal PICCs in E/VLBW infants to understand the incidences of various catheter-related complications and their risk factors to help form an empirical summary and evidence-based guidance for the improvement of practice.

**Methods:**

A retrospective study was conducted based on a 3-year practice of neonatal PICCs in E/VLBW infants. Neonatal health records were collected, including demographic characteristics, PICC placement data, and treatment information.

**Results:**

A total of 519 E/VLBW infants were included in this study. There were 77 cases of complications involving 72 infants with an overall incidence of 12.13%. The order of incidences of different complications from high to low was phlebitis (7.71%), malposition (3.66%), leakage (1.35%), pleural effusion (1.15%), central line-associated bloodstream infection (0.58%, 0.25/1,000d), and accidental removal (0.38%). Multivariate analysis revealed that the inserted vessel was an independent risk factor for PICC-associated complications (mainly phlebitis; *p *= 0.002). Neonatal PICCs inserted in the axillary vein were only one-tenth (*p *= 0.026) as likely to cause phlebitis as in the basilic vein, whereas when applied in the saphenous vein, neonatal PICCs were five times as likely to cause phlebitis (*p *= 0.000).

**Conclusion:**

E/VLBW infants might be more inclined to develop PICC-associated phlebitis. Catheters inserted in the axillary or basilic vein are preferred if possible.

## Background

Significant improvements in perinatal care have led to a substantial increase in the survival of very low birthweight (VLBW, birthweight <1,500 g) and extremely low birthweight (ELBW, birthweight <1,000 g) infants. The survival rate of ELBW infants in developed countries is significantly above 90%, whereas it has only reached 76% in China (1). Parenteral nutrition and medical treatment have made remarkable contributions to the improvement of neonatal care. They are also commonly required in the healthcare of E/VLBW infants to maintain their nutrition until sufficient volumes of enteral feeding can be tolerated, to avoid complications of hypoglycemia or sepsis in the early postnatal period, and to provide a potential nutrient reserve for subsequent catch-up growth. In such a case, a neonatal peripherally inserted central venous catheter (PICC) is frequently applied to facilitate the delivery of medication and nutrition when the long-term intravenous infusion is inevitable.

Recently, high-quality evidence has shown that the application of PICCs in neonates can avoid repetitive insertions and excessive use of medical resources, including transfusion consumables (2). However, it also requires high costs of catheter placement and maintenance, as well as causes various catheter-related complications, including central line-associated bloodstream infection (CLABSI), phlebitis, malposition, leakage, and even others deadly as perforation and thrombosis. Dong et al*.* reported a median cost of 198.8 Canadian dollars per pediatric patient per day (3), posing huge financial burdens to patients' households. The overall incidence of PICC-associated complications has been reported based on numerous studies to range from 16.4% to 37.2% (4–6), indicating a generally high occurrence, although with significant variation among countries. Despite the high cost and risk of complications, considering the profound significance of neonatal PICCs in supporting the lives of E/VLBW infants, neonatal intensive care unit (NICU) clinicians should make significant efforts to improve their quality in practice. Previous studies have greatly focused on identifying PICC-associated issues across the full-term neonatal population, but few have explored more sophisticated health care during neonatal PICC practice in E/VLBW infants. Therefore, we conducted a retrospective study of a 3-year clinical practice of neonatal PICCs in E/VLBW infants, aiming to understand the incidence of PICC-associated complications and their risk factors to help construct an empirical summary and evidence-based guidance for the improvement of practice.

## Materials and methods

### Study design

This retrospective cross-sectional study was conducted on the basis of a 3-year neonatal PICC clinical practice between January 1, 2017, and January 1, 2020, in a tertiary-level NICU at the West China Second University Hospital, Sichuan University. The hospital has 150 beds and provides centralized care for newborns with prematurity, low birthweight, or critical diseases across southwestern China. Our study was approved by the Ethics Committee of West China Second Hospital of Sichuan University (No. 2020098). Written consent was obtained from the parents of the patients, and PICC catheterization consent was signed. The information analyzed in the study will remain confidential and be used only for the scientific purposes presented here.

All E/VLBW infants who had at least one neonatal PICC catheter inserted and were admitted from January 1, 2017, to January 1, 2020, were eligible for enrollment. Cases reporting death, with missing critical information, or where PICCs were still in place when the infants were being transported between hospitals were excluded. The reason for excluding death cases was that these infants were most likely to have been diagnosed with serious basic diseases, adding to the risk of developing PICC-associated complications, which would be expected to cause a bias in our results. Any missing critical information, including data about PICC placement (type and number of PICC-associated complications, catheter tip positioning, indwelling time, inserted site, inserted vessel) or information about parenteral nutrition and medical treatment (e.g., antibiotics, calcium), would affect the accuracy of our results. Infants with neonatal PICCs *in situ* when transferred were excluded because it would be impossible to manage the quality control of PICC placement or monitor complications when they were in another hospital.

### Neonatal PICC placement and maintenance

A team consisting of neonatologists, nurses, and medical imaging technicians was in charge of the neonatal PICC placement and maintenance, and the entire procedure was implemented according to the Infusion Therapy Standards of Practice published by the Infusion Nurses Society in 2016 (7), with all nurses having obtained the qualification of advanced infusion practice. The details are as follows: (1) Before placement: The inserted site, inserted vessel, and the length of the catheter were evaluated. All infants were placed in the supine position. Infants with a catheter inserted in the head or neck had the length measured from the punctured point along the vein to the right nipple with the head inclined to one side; infants with a catheter inserted in the upper extremity had the length measured from the punctured point through the right sternoclavicular joint down to the second intercostal space with a 90° abduction of the upper extremity; infants with a catheter inserted in the lower extremity had the length measured from the punctured point along the groin to the midpoint of umbilicus and xiphoid with a natural abduction of the lower extremity. (2) During placement: The inserted site was cleansed with normal saline and disinfected twice with 10% povidone-iodine. According to maximal barrier precautions, all operators and assistants were equipped with surgical caps, masks, sterile gowns, sterile gloves, and sterile full-body drapes throughout the process. All catheters were of polyurethane 1.9F type, produced by BD company. (3) After placement: Bedside x-rays were performed within 1 h after PICC placement to locate the catheter tip. The superior junction of the superior vena cava and right atrium was considered an ideal central position of the catheter tip for PICCs inserted in the head, neck, or upper extremity, and the inferior vena cava above the diaphragm was the central position of the catheter tip in cases of lower extremity insertion. Adjustments in catheter placement were made according to the x-ray radiographs in cases of noncentrally positioned catheter tips (4). Maintenance: All catheters were sealed with 1.0 IU/mL heparin at the end of the infusion or every 6 h in the case of continuous infusion. The transparent dressing was replaced every 7 days or immediately once contaminated or when its integrity was damaged. Any procedure concerning blood product transfusion or collection of blood samples was forbidden through the catheter, and measurement of noninvasive blood pressure was not allowed through the inserted extremity.

### Data collection

The entire neonatal PICC application procedure in each case was tracked by researchers. Two researchers were in charge of the quality control of neonatal PICC placement and maintenance. One took daily records of the detailed conditions of each included patient using specially designed forms, and the other reviewed the records to ensure data accuracy. The neonatal health records of all patient were retrieved, including demographic characteristics (e.g., gender, gestational age, age at catheterization, and weight at catheterization), data about PICC placement (type and number of PICC-associated complications, catheter tip positioning, indwelling time, inserted site, inserted vessel), and information about parenteral nutrition and medical treatment (e.g., antibiotics, calcium). In particular, the ideal central positioning in our study was defined as the lower segment of the superior vena cava at or near the cavoatrial junction for upper body insertion sites and the inferior vena cava above the level of the diaphragm for lower body insertion sites (7). Furthermore, two conditions of the “noncentral position” were considered in our study. The first was when a catheter tip moved from the central position to the noncentral-positioned vena cava or great veins (e.g., brachiocephalic vein, iliac vein) at the final use stage of neonatal PICC, mainly due to the infants' escalating bodyweight, length, or physical activity. The second condition was when the catheter tip was moved somewhere other than the vena cava or great veins, specifically termed “malposition.”

### Outcomes

The primary outcome of this study was the incidence of PICC-associated complications in E/VLBW infants, and the secondary outcome was the determined risk factors associated with these complications. Specifically, the complications in this study were CLABSI, phlebitis, malposition, leakage, pleural effusion, thrombosis, and accidental removal. CLABSI was diagnosed by a positive peripheral blood culture of neonates during PICC or within 48 h after catheter removal. Phlebitis was defined as inflammation of the vein with typical symptoms including erythema, pain, swelling, or palpable venous cord. Malposition was diagnosed using x-rays when the tip of the catheter was not in the vena cava or great veins, such as when it was too deep, folded, or prolapsed. Leakage was when fluid was detected leaking into the soft tissue around the catheter or body cavity. The pathogenesis of pleural effusions mainly involves hyperosmolar endothelial damage that leads to increased vascular permeability, and thrombosis is mainly due to friction between catheter and vessel; both were diagnosed through ultrasound. Accidental removal refers to the circumstance when the catheter is removed unintentionally during dressing changes or other care progress.

### Data analysis

SPSS 22.0 software (IBM Corp, Chicago, IL, USA) was employed for statistical analyses. Numbers, medians, maxima, minima, and percentages were used to describe the characteristics and incidence of PICC-associated complications in E/VLBW infants. Both the chi-squared test and the Mann–Whitney *U* test were used for univariate analysis to explore significant predictors of PICC-associated complications, where differences among groups categorized by percentage were assessed by the chi-squared test, and analysis of non-normally distributed variables was performed by the Mann–Whitney *U* test. Significant predictors identified in the univariate analysis (“inserted site” and “inserted vessel”) were included as candidate predictors in a binary logistic regression for further multivariate analysis, aiming to explore the risk factors of PICC-associated complications. Inspired by the remarkably high occurrence of phlebitis in E/VLBW infants derived from our analysis, we conducted a second regression to explore its risk factors, incorporating the same candidate variables identified as significant in the prior univariate analysis. Categorical variables were processed as dummy variables and continuous variables as original values in these two forward selection regressions. *p* ≤ 0.05 was considered statistically significant.

## Results

A total of 526 cases were enrolled in our study, of which 7 were excluded (1 due to death, 3 due to inadequate information about PICCs, and 3 because parents refused to take peripheral blood culture tests), leaving 519 cases qualified. These comprised 273 (52.6%) boys and 246 (47.4%) girls with a median gestational age of 29.41 weeks (23–39 weeks). The median age at catheterization was 1.36 days (0–47 days), and the median weight at catheterization was 1,245.22 g (590–1,490 g). A total of 224 (43.2%) infants were inserted with the catheter tip in a central position, and 295 (56.8%) had a noncentral position. The median indwelling time was 12.84 days (1–60 days). Most infants had the inserted site as the upper extremity (464, 89.4%), and the most common inserted vessel was the basilic vein (291, 56.1%). As for medical treatment and parenteral nutrition, 429 (82.7%) infants were infused with β-lactam antibiotics, and the remaining 90 (17.3%) received vancomycin. A total of 238 (45.9%) infants had calcium gluconate infused, and almost all had parenteral nutritional support (517, 99.6%). Details are given in Table 1.

**Table 1:** Characteristic of ELBW/VLBW infants (*n* = 519).

Items	Numbers (%)/median (min, max)
Gender	
Boy	273 (52.6)
Girl	246 (47.4)
Gestational age (weeks)	29.41 (23.0, 39.0)
Age at catheterization (days)	1.36 (0.0, 47.0)
Catheter tip positioning	
Central	224 (43.2)
Noncentral	295 (56.8)
Indwelling time (days)	12.84 (1.0, 60.0)
Inserted site	
Upper extremity	464 (89.4)
Lower extremity	32 (6.2)
Head	20 (3.9)
Neck	3 (0.6)
Inserted vessel	
Basilic vein	291 (56.1)
Scalp vein	20 (3.9)
Jugular vein	3 (0.6)
Axillary vein	129 (24.9)
Cephalic vein	44 (8.5)
Saphenous vein	32 (6.2)
Antibiotics	
β-Lactams	429 (82.7)
Vancomycin	90 (17.3)
Calcium (calcium gluconate)	
Without	238 (45.9)
With	281 (54.1)
Parenteral nutrition	
Without	2 (0.4)
With	517 (99.6)

ELBW/VLBW, extremely low birthweight/very low birthweight.

There were 77 cases of complications involving 72 infants, with an overall incidence of 12.13%. Five infants were diagnosed with multiple complications: one with phlebitis and accidental removal, two with phlebitis and malposition, one with phlebitis and leakage, and one with pleural effusion and CLABSI. The order of incidence of complications from high to low was phlebitis (7.71%), malposition (3.66%), leakage (1.35%), pleural effusion (1.15%), CLABSI (0.58%, 0.25/1,000 d), accidental removal (0.38%), and thrombosis (0%), as listed in Table 2.

**Table 2:** Incidence of PICC-associated complications in ELBW/VLBW infants (*n *= 519).

Complications	Number	Incidence (%)
Phlebitis	40	7.71
Malposition	19	3.66
Leakage	7	1.35
Pleural effusion	6	1.15
CLABSI	3	0.58, 0.25/1,000 d
Accidental removal	2	0.38
Thrombosis	0	0.00
Total	72	12.13

PICC, peripherally inserted central venous catheter; ELBW/VLBW, extremely low birthweight/very low birthweight; CLABSI, central-line associated-bloodstream infection.

Univariate analysis suggested the inserted site (*χ*^2^ = 13.349, *p *= 0.004) and inserted vessel (*χ*^2^ = 20.552, *p *= 0.001) as significant predictors of PICC-associated complications in E/VLBW infants, whereas no significance was detected in the variables of gender, age at catheterization, weight at catheterization, indwelling time, catheter tip positioning, use of antibiotics, use of calcium, or use of parenteral nutrition, as presented in Table 3. Multivariate analysis indicated that inserted vessel was an independent risk factor for PICC-associated complications, with an odds risk (OR) of 3.487 in the saphenous vein compared to the basilic vein (*p *= 0.002; Table 4). Further binary logistic regression showed that the inserted vessel was mainly associated with phlebitis, suggesting that a neonatal PICC inserted into the axillary vein was a protective factor for phlebitis (OR = 0.100, *p *= 0.026), while the saphenous vein appeared to be a risk factor (OR = 5.031, *p *= 0.000) (Table 5).

**Table 3:** Univariate analysis of PICC-associated complications in ELBW/VLBW infants.

Variable	Complication		*χ*^2^/Z	*p*
	Without (*n* = 447) %/mean (min, max)	With (*n* = 72) %/mean (min, max)		
Gender				
Boy	228 (51.0)	45 (62.5)		
Girl	219 (49.0)	27 (37.5)	3.285^a^	0.070
Age at catheterization (days)	1.35 (0.0, 47.0)	1.48 (0.0, 36.0)	−0.100^b^	0.920
Weight at catheterization (g)				
<1,000	68 (15.2)	9 (12.5)		
1,000–1,500	379 (84.8)	63 (87.5)	0.361^a^	0.548
Indwelling time (days)	13.13 (1.0, 60.0)	10.86 (2.0, 45.0)	−1.000^b^	0.317
Inserted site				
Upper extremity	407 (91.1)	57 (79.2)		
Lower extremity	21 (4.7)	11 (15.3)		
Head	16 (3.6)	4 (5.6)		
Neck	3 (0.7)	0 (0.0)	13.349^a^	0.004*
Inserted vessel				
Basilic vein	253 (56.6)	38 (52.8)		
Scalp vein	16 (3.6)	4 (5.6)		
Jugular vein	3 (0.7)	0 (0.0)		
Axillary vein	120 (26.8)	9 (12.5)		
Cephalic vein	34 (7.6)	10 (13.9)		
Saphenous vein	21 (4.7)	11 (15.3)	20.552^a^	0.001*
Catheter tip positioning				
Central	199 (44.5)	25 (34.7)		
Noncentral	248 (55.5)	47 (65.3)	2.426^a^	0.119
Antibiotics				
β-Lactams	374 (83.7)	55 (76.4)		
Vancomycin	73 (16.3)	17 (23.6)	2.293^a^	0.130
Calcium (calcium gluconate)				
Without	206 (46.1)	32 (44.4)		
With	241 (53.9)	40 (55.6)	0.067^a^	0.795
Parenteral nutrition				
Without	2 (0.4)	0 (0.0)		
With	445 (99.6)	72 (100.0)	0.323^a^	0.570

PICC, peripherally inserted central venous catheter; ELBW/VLBW, extremely low birthweight/very low birthweight.

^a^ *χ*^2^ test; ^b^Mann–Whitney *U* test; *p < 0.05.

**Table 4:** Multivariate analysis of PICC-associated complications in ELBW/VLBW infants.

Variable	*B*	SE	*p*	*OR*	95%CI
Inserted vessel: basilic vein as the reference	–	–	0.003*	–	–
Scalp vein	0.510	0.585	0.384	1.664	0.528, 5.244
Jugular vein	−19.307	23,205.422	0.999	0.000	0.000, -
Axillary vein	−0.694	0.387	0.073	0.499	0.234, 1.066
Cephalic vein	0.672	0.400	0.093	1.958	0.895, 4.285
Saphenous vein	1.249	0.411	0.002*	3.487	1.559, 7.802
Constant	−1.896	0.174	0.000*	0.150	–

PICC, peripherally inserted central venous catheter; ELBW/VLBW, extremely low birthweight/very low birthweight.

* *p *< 0.05.

**Table 5:** Risk factors of PICC-associated phlebitis in ELBW/VLBW infants.

Variable	*B*	SE	*p*	*OR*	95%CI
Inserted vessel: basilic vein as the reference	–	–	0.001*	–	–
Scalp vein	0.357	0.779	0.647	1.429	0.310, 6.577
Jugular vein	−18.649	23,205.422	0.999	0.000	0.000, –
Axillary vein	−2.298	1.029	0.026*	0.100	0.013, 0.755
Cephalic vein	0.889	0.470	0.059	2.432	0.968, 6.115
Saphenous vein	1.616	0.454	0.000*	5.031	2.067, 12.244
Constant	−2.554	0.227	0.000*	0.078	–

PICC, peripherally inserted central venous catheter; ELBW/VLBW, extremely low birthweight/very low birthweight.

* *p* < 0.05.

## Discussion

E/VLBW infants are born with thinner and flimsier vessels, extremely immature immunity, and more fragile skin barriers, making it a huge challenge for NICU clinicians to insert and maintain a neonatal PICC (8). Since few studies have focused on PICC-associated complications in E/VLBW infants, we conducted this study to help fill this gap and make significant progress in neonatal PICC clinical practice.

The commonest PICC-associated complication in E/VLBW infants reflected in our study was phlebitis, and its independent risk factor was the saphenous vein as the inserted vessel, whereas the protective factor was the axillary vein as the inserted vessel. Phlebitis was a common vascular access complication, and its incidence in E/VLBW infants in this study was 7.71%. This is similar to another study that reported a 12% incidence in low-birthweight infants (9) but much higher than the rate of 0.6%–3.5% reported in the full-term neonatal population (10, 11). This can be explained as phlebitis is mainly caused by mechanical stimulation (12); thus, it would be preferable to use 1 Fr catheters to reduce the risk of mechanical stimulation when the vein is small, especially the femoral vein. However, the neonatal PICC placement team in this study always used 1.9 Fr catheters, which might increase long-term friction between the catheter and vascular wall. In our review of previous literature, early removal of catheters is thought to avoid further adverse consequences from this issue. However, Igarashi et al*.* reported that up to 34.5% of PICCs were removed due to phlebitis (13), revealing that this seemingly incisive measure is possibly at the expense of a generally reduced application of neonatal PICCs. Our study found that E/VLBW infants might be more inclined to experience PICC-associated phlebitis, and we hope this will attract more attention from NICU clinicians to address this issue. Moreover, we suggest that more detailed evaluations be made in the ordinary maintenance of neonatal PICCs in E/VLBW infants, and effective measures to prevent and manage PICC-associated phlebitis should be explored in future studies.

Numerous studies have tried to identify the risk factors for PICC-associated complications in neonates, and whether inserting into the upper or lower extremity is likely to cause a higher risk of complications is one of the most concerning issues. Hoang et al. found that neonatal PICCs applied in the lower extremity showed a lower incidence of CLABSI than in the upper extremity, suggesting that parenteral nutrition can be better administered through catheters inserted in the lower extremity (14). Nonetheless, Wrightson et al*.* and Bashir et al. reported no significant difference in the incidences of PICC-associated complications between the upper and lower extremities (15, 16). No clear evidence on this issue existed until a meta-analysis was published (17), which demonstrated that neonatal PICCs in the upper extremity were more inclined to cause malposition but had a lower risk of thrombosis than in the lower extremity. However, this evidence was not strong enough, with all the included studies being cross-sectional. In our study, the results from the univariate analysis showed significantly different incidences of complications among the upper and lower extremities and other inserted sites, but the “inserted site” as a predictor was filtered out after it was included in the regression model together with another variable of “inserted vessel.” We speculated that this was due to the intersection between the two variables, and it was thought more instructive to explore the influence of different inserted vessels on PICC-associated complications in neonates rather than different inserted sites such as upper or lower extremities. Our multivariate analysis showed that inserted vessel was the only determining risk factor for PICC-associated complications, and it was further proved to be mainly associated with phlebitis. This was similar to the results acquired by Pet et al*.*, who reported that neonatal PICCs applied in upper arm vessels were associated with a decreased incidence of phlebitis (4). Moreover, Panagiotounakou et al*.* found that premature neonates with PICCs in the axillary vein had one-twelfth the chance to develop complications compared with those with PICCs in the basilic vein (18). This is consistent with our findings that PICCs in the axillary vein were one-tenth as likely to cause phlebitis as were PICCs in the basilic vein, whereas PICCs in the saphenous vein were five times as likely to cause this complication. This may be because compared with the axillary vein, the saphenous vein has a smaller inner diameter and a long distance from the inserted site to the endpoint of the central position (19), and the contact area of the catheter and the vessel is larger, leading to more frequent friction and thus a higher risk of phlebitis caused by mechanical stimulation. Therefore, based on the results of previous studies and ours, we suggest that the neonatal PICC should be inserted in the largest possible vein, always keeping in mind that the diameter of the catheter should not exceed one-third of the diameter of the vessel.

According to this study, other comparatively prominent complications also deserve focus, such as malposition and leakage, ranking second and third, respectively. Catheter tip malposition is a PICC-associated complication that can lead to deadly adverse consequences and is also closely associated with leakage and perforation. The rate of malposition in recent research was reported as 56.0% (20), leakage as 2.0% (10), and pleural effusion as 0.51% (16, 21). In E/VLBW infants, the rates of malposition, leakage, and pleural effusion were found to be 3.66%, 1.35%, and 1.15%, respectively, in this study. During insertion, the catheter tip may move into noncentral veins where rapid dilution of hypertonic drugs cannot be effectively achieved, and this chemical stimulation can continuously corrode vascular walls, leading to vessel perforation, leakage of infusion drugs, pleural effusion, pericardial effusion, or even fatal cardiac tamponade in severe cases (22). Bundle strategies such as enhancement of catheter fixation, daily monitoring of the exposed length of the catheter, and regular x-rays to locate the catheter tip position are highly recommended to decrease deadly complications.

### Limitations

This study has some limitations. First, the incidence of CLABSI was unexpectedly low, which we believe was because it can only be diagnosed with a positive peripheral blood culture during catheter indwelling. However, the blood culture test was not applied in every case due to household financial difficulties, despite the patients’ obvious clinical signs, which could lead to a false-negative result. Second, performing regular x-rays for catheter tip positioning is outdated, and ultrasound has now proven superior, but it was reasonable at that time considering that our study is based on a 3-year clinical practice between 2017 and 2020. Third, disinfection was performed with 10% povidone-iodine in this study, but the latest guideline has recommended chlorhexidine in alcohol or aqueous solution for disinfection, which should be highlighted. Finally, being a retrospective study, the sample size was not large enough for the cases to be divided into two groups—ELBW infants and VLBW infants—for data analysis. Thus, more infants will continue to be included in this study until it is sufficient for hierarchical analysis.

## Conclusion

This study presented a review of a 3-year clinical practice of neonatal PICCs in E/VLBW infants. The results showed that the commonest PICC-associated complication in E/VLBW infants was phlebitis, with other prominent complications of malposition and leakage ranking second and third, respectively. PICCs were only one-tenth as likely to cause phlebitis in the axillary vein and five times as likely to cause it in the saphenous vein, both compared with insertion in the basilic vein. We sincerely suggest that neonatal PICC should be inserted in the axillary or basilic vein in E/VLBW infants whenever possible.

## Data Availability

The original contributions presented in the study are included in the article/Supplementary Material, further inquiries can be directed to the corresponding author/s.
